# Case Report: Neuronal Intranuclear Inclusion Disease With Oromandibular Dystonia Onset

**DOI:** 10.3389/fneur.2021.618595

**Published:** 2021-02-11

**Authors:** Wei-Ping Deng, Zhao Yang, Xiao-Jun Huang, Jing-Wen Jiang, Xing-Hua Luan, Li Cao

**Affiliations:** ^1^Department of Neurology, Rui Jin Hospital, Shanghai Jiao Tong University School of Medicine, Shanghai, China; ^2^Department of Neurology, Shanghai Jiao Tong University Affiliated Sixth People's Hospital, Shanghai, China

**Keywords:** neuronal intranuclear inclusion disease, oromandibular dystonia, skin biopsy, NOTCH2NLC gene, clinical manifestations

## Abstract

**Background:** Neuronal intranuclear inclusion disease (NIID) is a rare neurodegenerative disease. Because of variable clinical manifestations, NIID was often misdiagnosed. According to published case reports, the common clinical manifestations of NIID include dementia, muscle weakness, autonomic impairment, sensory disturbance, rigidity, ataxia convulsions, etc. However, no cases of oromandibular dystonia were mentioned.

**Case Presentation:** We describe a case of a 58-year-old woman presenting with mouth involuntary chewing initially. She started to show hand tremors, ataxia, and walking instability until 2 years later. Diffusion-weighted imaging showed high intensity signal along the corticomedullary junction. Fluid-attenuated inversion recovery imaging showed white matter hyperintensity. Electromyography (EMG) indicated peripheral nerve degeneration. Neuropsychological testing showed memory loss. Finally, skin biopsy and GGC repeat expansions in the *NOTCH2NLC* (Notch 2 N-terminal like C) gene confirmed the diagnosis of NIID.

**Conclusion:** This case demonstrated that oromandibular dystonia could be the first symptom of NIID. This case report provides new characteristics of NIID and broadens its clinical spectrum.

## Introduction

Neuronal intranuclear inclusion disease (NIID) is a slowly progressive neurodegenerative disease characterized by eosinophilic hyaline intranuclear inclusions in the central, peripheral, and autonomic nervous systems (1). Head MRI shows high intensity signal along the corticomedullary junction on diffusion-weighted image and white matter hyperintensity on fluid-attenuated inversion recovery images (2, 3). Skin biopsy reveals the presence of eosinophilic hyaline intranuclear inclusions to confirm the diagnosis (4). The clinical manifestations of NIID are highly heterogeneous, including dementia, muscle weakness, autonomic impairment, sensory disturbance, rigidity, ataxia, and convulsions (5). However, oromandibular dystonia is rarely seen. Here, we described a patient presenting with mouth involuntary chewing as the first symptom and had been misdiagnosed several times. She was finally diagnosed as NIID based on the skin pathology and genetic analysis. This case report describes a rare clinical manifestation of NIID, which will contribute to a better understanding of the clinical characteristics of NIID for better diagnosis in the future.

## Case Presentation

A 58-year-old woman was admitted to our hospital with a 2-year history of mouth involuntary chewing and a 1-month history of hand tremor. She presented with mouth involuntary chewing initially 2 years ago. It was easy to bite the tongue when speaking. Symptoms gradually worsen as the course of the disease progressed. She began to have vague speech and a sense of fatigue. She had excessive saliva and complaining of chronic constipation. Two years later, she had shaky hands, difficulty in swallowing, and walking. During the course of the disease, the patient had no other discomforts, such as headache, dizziness, limb convulsions, etc. She denied a history of chronic diseases, such as high blood pressure, diabetes, and heart disease. There was no family history of neurological problems. A neurologic examination found vague speech, involuntary and repetitive jaw deviation, and chewing. Muscle strength was evaluated as 4/5 in the hands and as 4^+^/5 in the lower limbs on the Medical Research Council (MRC) scale. Hypoesthesia to pinprick in the left arm was detected. Deep tendon reflexes could not be elicited. Resting and postural tremors in the hands were noted, and gait was slightly unsteady. The finger-to-nose test was positvie on the left side and the Romberg's sign was also positive.

**Figure 1:**
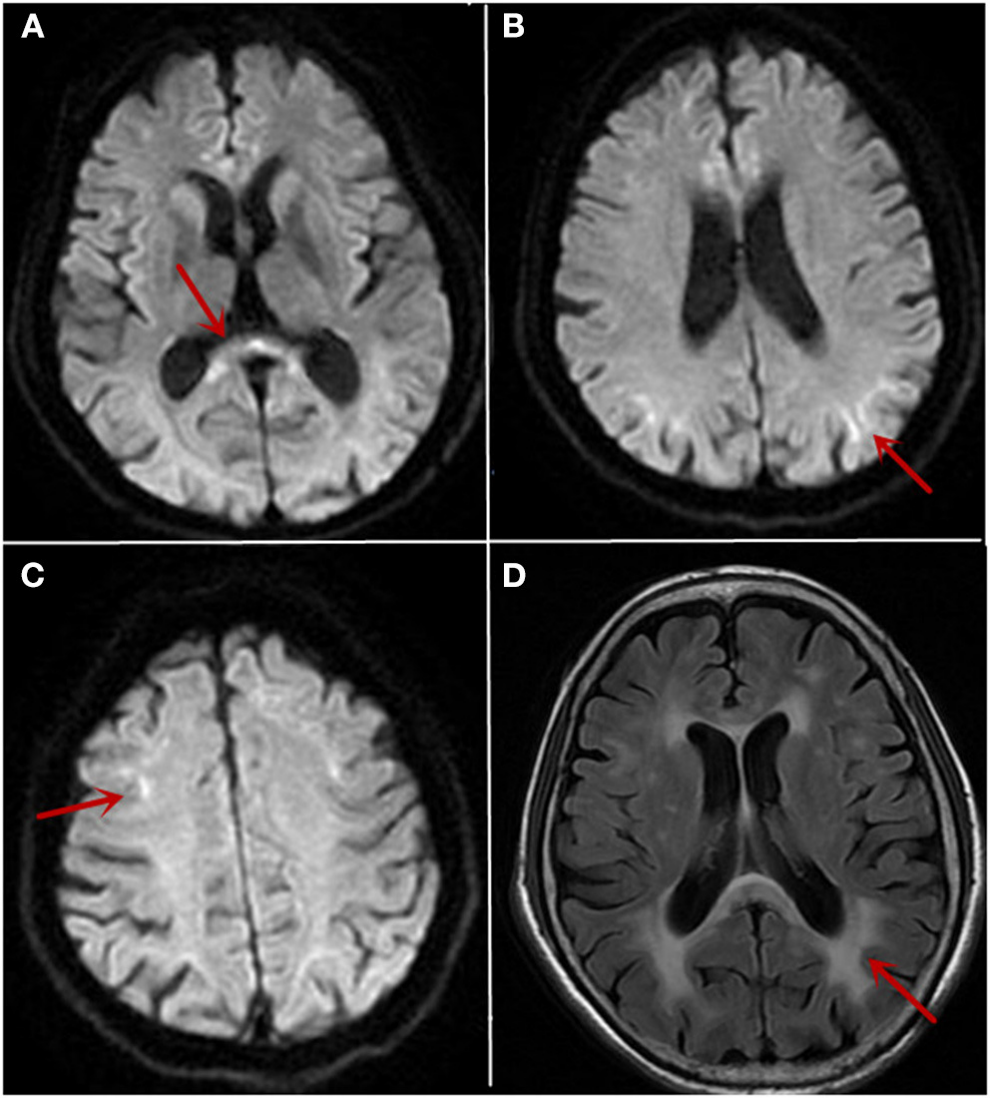
The cranial MRI findings on 10/26/2019 and 12/12/2019. MRI showed high intensity signal in the corpus callosum (**A**, arrow) and along the corticomedullary junction on diffusion-weighted image (**B,C**, arrow). White matter hyperintensity on fluid-attenuated inversion recovery images (**D**, arrow) was also noticed.

**Figure 2:**
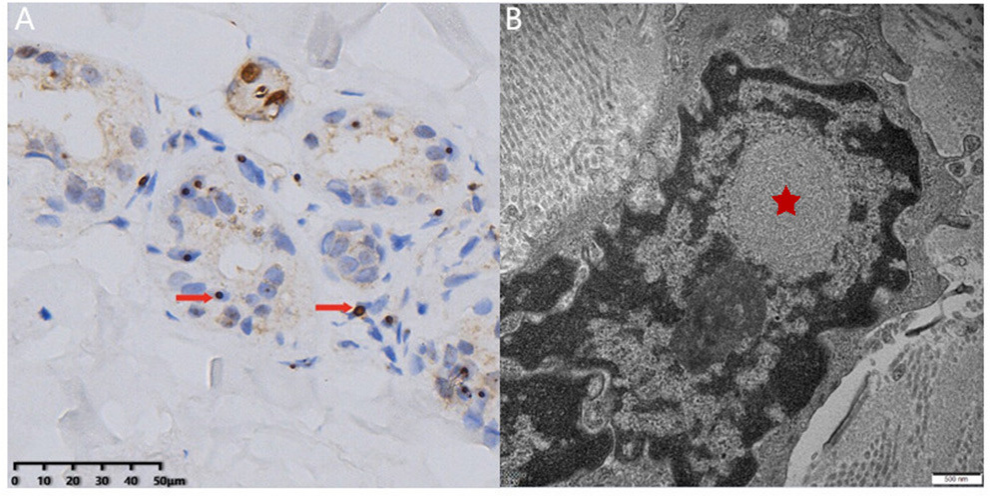
Skin biopsy examination. Light microscopy revealed round-shaped p62-positive intranuclear inclusions in the sweat gland cells (**A**, arrow). Electron microscopy showed dense filament material without membrane (**B**, star) (bar = 500 nm).

After admission, relevant examinations were carried out. No abnormality was revealed by blood routine, routine urine and stool testing, biochemical indicators, coagulation function, thyroid function, glycosylated hemoglobin, and tumor markers. The titer of anti-ANA was 1:100, and anti-Ro-52 was positive (+). Anti-double-stranded DNA, anti-CCP, anti-SSA, ANCA, and other autoantibody tests were negative. A cerebrospinal fluid examination showed an intracranial pressure of 85 mm H_2_O, nucleated cell count of 1 × 10^6^ cells/L, total protein level of 484.20 mg/L, glucose level of 3.70 mmol/L, and chloride level of 129.00 mmol/L. Autoimmune encephalitis antibody tests of the cerebrospinal fluid were negative. Electromyography (EMG) showed that the motor and sensory conduction velocity was generally slower than normal, with an average conduction velocity of 40 or 35 m/s, respectively. The shortest latency of the bilateral ulnar nerve and the tibial nerve F wave was prolonged. Head MRI showed high intensity signal along the corticomedullary junction on diffusion-weighted image and white matter hyperintensity on fluid-attenuated inversion recovery images (Figure 1). The neuropsychological test of the patient revealed mild cognitive decline with a Mini-Mental State Examination (MMSE) score of 24 and a Montreal Cognitive Assessment (MoCA) score of 20, especially in language and executive functions. Skin biopsy found round-shaped p62-positive intranuclear inclusions in the sweat gland cells, fibroblast cells, and adipocytes (Figure 2A). Dense filament material without membrane was observed by electron microscope (Figure 2B). Genetic examination using GC-rich PCR (GC-PCR) and repeat-primed PCR (RP-PCR) found the repeat expansion as a saw-tooth pattern (Supplementary Figure 1). The patient had 115 GGC repeats in the 5′ UTR of the *NOTCH2NLC* (Notch 2 N-terminal like C) gene. Genetic testing for FMR1 CGG repeat expansion was negative. At the same time, her son also had performed the genetic examination, and it was normal. With the evidence of characteristic imaging support, optimal genetic testing, and definite pathological diagnosis, it was diagnosed as NIID.

## Discussion

Neuronal intranuclear inclusion disease is a group of degenerative diseases characterized by regional neuron loss and the presence of eosinophilic intranuclear inclusion in the glial cells and neurons (1). At the same time, the pathology could show positive staining of p62, ubiquitin, and small ubiquitin-like modifier-1 (SUMO-1) of the eosinophilic intranuclear inclusions (5, 6). Dense filament material without membrane could be observed by electron microscope.

In 2016, Sone et al. (1) summarized the clinical characteristics of 38 cases of sporadic NIID. The adult-onset NIID often had dementia or limb weakness as early symptoms. The most common clinical manifestation of sporadic adult NIID was dementia, and most patients had miosis (94.4%). Adult NIID could also present peripheral nerve damage including muscle weakness (27%) and sensory disturbance (28.6%). Other symptoms of autonomic nervous system involvement included bladder dysfunction (33.3%). In addition, ataxia is a common manifestation, which occurs in 52.8% of sporadic adult-onset NIID in patients. Sone et al. (1) had found some patients presenting with subacute progressive encephalitis. Other symptoms were also reported, including tremor, convulsion, abnormal mental behavior, rigidity, etc. Dystonia is a rare clinical manifestation of NIID. According to previous research reports, NIID patients could present with dopa-responsive dystonia. Six cases of NIID had been described where early parkinsonism was a predominant clinical feature (7). However, no cases with oromandibular dystonia were mentioned. In this case, a 58-year-old woman presented with mouth involuntary chewing as the first symptom. As the course of the disease progressed, the patient gradually showed vague speech and a sense of fatigue. Compared with the previously reported typical manifestations, the patient had no obvious dementia, ataxia, tremor, convulsion, and other typical manifestations in the initial stage. Chewing, swallowing, and speech were frequently impaired in oromandibular dystonia, altering quality of life and social relationships (8). She started to show hand tremors, ataxia, and walking instability until 2 years later. The neuropsychological test of the patient revealed mild cognitive decline during hospitalization. EMG indicated potential peripheral nerve damage. Because of the atypical symptoms of the disease or insufficient understanding of the disease in the early stage, it was difficult to make early diagnosis.

In patients with NIID, MRI (1, 2) showed diffuse symmetric white matter hyperintensity around the lateral cerebral ventricle on fluid-attenuated inversion recovery images and high intensity signal along the corticomedullary junction on diffusion-weighted image, which was known as “cortical linear sign.” The study found that over a period of 10 years, the imaging of NIID patients suggested that cortical linear sign existed continuously at the corticomedullary junction of diffusion-weighted imaging (DWI) (9, 10), and that the lesion was mainly limited to the frontal lobe at the early stage of the disease. With the progress of the disease course, the high signal intensity of DWI gradually developed from the frontal lobe to the occipital lobe rather than to the deep white matter (11, 12). This feature may accompany the entire course of the disease and may be an important basis for distinction from other diseases. We also thought about NIID by following up this patient for 2 months and found a persistent “cortical linear sign” on DWI. Finally, the diagnosis was confirmed by further perfecting the pathological examination and gene examination.

Neuronal intranuclear inclusion disease is similar to other neurodegenerative diseases, and there is no special recommended treatment. Symptomatic treatment may improve the quality of the patient's life and delay the progress of some symptoms, such as Parkinson's syndrome, epilepsy, and dementia. Identifying and intervening of patients with suspected NIID as soon as possible may be the key to diagnosis and treatment.

In summary, NIID is involved in a wide range of parts, and the heterogeneity of symptoms is obvious. The clinical heterogeneity of NIID increases the difficulty of our diagnosis. Dementia, ataxia, and limb weakness are more common clinical manifestations in NIID patients. High intensity signal along the corticomedullary junction is of high value for diagnosis. This case provides that oromandibular dystonia may be the initial symptom of NIID. In the future, more international studies may find more clinical symptoms of NIID and clarify the pathogenesis of NIID, so as to develop a more effective treatment for this disease.

## Data Availability Statement

The raw data supporting the conclusions of this article will be made available by the authors, without undue reservation.

## Ethics Statement

The studies involving human participants were reviewed and approved by Rui Jin Hospital Ethics Committee, Shanghai Jiao Tong University School of Medicine. The patients/participants provided their written informed consent to participate in this study. Written informed consent was obtained from the individual(s) for the publication of any potentially identifiable images or data included in this article.

## Author Contributions

W-PD and ZY: data collection and drafting the manuscript. X-JH and J-WJ: data evaluation and manuscript revision. X-HL: pathological test and manuscript revision. LC: funding, data collection and evaluation, supervision, manuscript revision, and final approval. All authors contributed to the article and approved the submitted version.

## Conflict of Interest

LC is in charge of the National Natural Science Foundation of China (81870889 and 82071258). The remaining authors declare that the research was conducted in the absence of any commercial or financial relationships that could be construed as a potential conflict of interest.
