# Supplementary figures and images for: Case Report: Neuronal Intranuclear Inclusion Disease With Oromandibular Dystonia Onset
Source: Front Neurol. 2021 Feb 11;12:618595. doi: 10.3389/fneur.2021.618595 (PMC7928273; doi:10.3389/fneur.2021.618595)

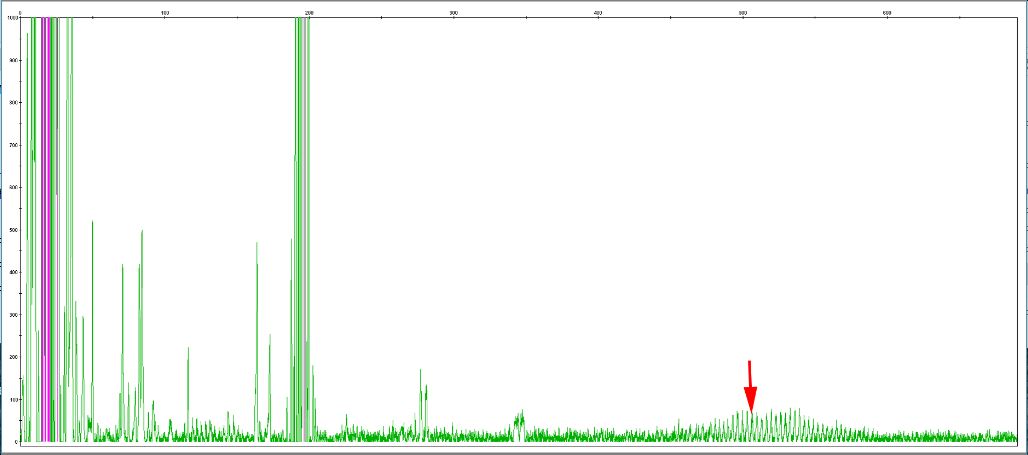

Supplement: Supplementary file 1 [file Image_1.JPEG]
