# Supplementary material for: Case Report: Neuronal Intranuclear Inclusion Disease With Oromandibular Dystonia Onset
Source: Front Neurol. 2021 Feb 11;12:618595. doi: 10.3389/fneur.2021.618595 (PMC7928273; doi:10.3389/fneur.2021.618595)
Supplement: Supplementary file 2 [file Table_1.DOCX]

**Figure S1** Repeat length in NOTCH2NLC was detected by fluorescence amplicon length analysis. A gain of 115 repeat units was found (arrow).
